# miR-1285-3p Controls Colorectal Cancer Proliferation and Escape from Apoptosis through DAPK2

**DOI:** 10.3390/ijms21072423

**Published:** 2020-03-31

**Authors:** Lidia Villanova, Chiara Barbini, Cristina Piccolo, Alessandra Boe, Ruggero De Maria, Micol Eleonora Fiori

**Affiliations:** 1 Department of Oncology and Molecular Medicine, Istituto Superiore di Sanità, 00161 Rome, Italy; 2 Core Facilities, Istituto Superiore di Sanità, 00161 Rome, Italy; 3 Institute of General Pathology, Catholic University of the Sacred Heart and Gemelli Polyclinic, 00168 Rome, Italy

**Keywords:** colorectal cancer, microRNAs, LNAs, apoptosis, cell cycle, DAPK2, cancer stem cells

## Abstract

MicroRNAs are tiny but powerful regulators of gene expression at the post-transcriptional level. Aberrant expression of oncogenic and tumor-suppressor microRNAs has been recognized as a common feature of human cancers. Colorectal cancer represents a major clinical challenge in the developed world and the design of innovative therapeutic approaches relies on the identification of novel biological targets. Here, we perform a functional screening in colorectal cancer cells using a library of locked nucleic acid (LNA)-modified anti-miRs in order to unveil putative oncogenic microRNAs whose inhibition yields a cytotoxic effect. We identify miR-1285-3p and further explore the effect of its targeting in both commercial cell lines and primary colorectal cancer stem cells, finding induction of cell cycle arrest and apoptosis. We show that DAPK2, a known tumor-suppressor, is a novel miR-1285 target and mediates both the anti-proliferative and the pro-apoptotic effects of miR-1285 depletion. Altogether, our findings uncover a novel oncogenic microRNA in colorectal cancer and lay the foundation for further studies aiming at the development of possible therapeutic strategies based on miR-1285 targeting.

## 1. Introduction

Colorectal cancer (CRC) is the third leading cause of cancer death in developed countries [1]. Although several therapeutic approaches are currently available, ranging from conventional chemotherapy and radiotherapy to the more recent targeted therapies, the prognosis of patients with metastatic colorectal cancer remains poor. 

MicroRNAs (miRNAs) are small evolutionarily conserved non-coding RNAs (~22 nt in length) that act as post-transcriptional regulators of gene expression [2]. MiRNA expression is dysregulated in cancer owing to chromosomal rearrangements, point mutations, epigenetic alterations, changes in transcription factors activity or abnormalities in the miRNA biogenesis machinery [3]. Aberrantly expressed microRNAs are involved in multiple processes underlying colorectal carcinogenesis, such as cell proliferation, evasion of apoptosis, tumor invasion, angiogenesis, microsatellite instability and decreased drug sensitivity [4]. Among the microRNA-based anti-cancer therapies, chemically modified antisense oligonucleotides (anti-miRs) are designed to bind directly to the mature strand of the targeted miRNA and represent the most successful strategy to induce a functional blockade of oncogenic miRNAs [5]. Locked nucleic acid (LNA)-modified anti-miRs are chemically locked by a bridge that connects the 2′-oxygen and 4′-carbon in a ribonucleotide, conferring an unprecedented hybridization affinity and stability to nuclease degradation. 

Intrinsic apoptosis represents a first-line defense strategy against transformed phenotypes and escape from apoptosis was classified as a hallmark of cancer, being responsible for therapy failure and resistance [6,7]. DAPK2 is a Ca^2+^/Calmodulin (CaM)-regulated serine threonine kinase belonging to the DAP-kinase family of proteins, which function as positive mediators of apoptosis and autophagy [8]. First described as a pro-apoptotic factor, DAPK2 induces the morphological changes characteristic of apoptosis, such as membrane blebbing and chromatin condensation [9,10]. Moreover, DAPK2 contributes to the execution of anoikis (i.e., apoptosis induced by cell detachment from the extracellular matrix) in non-malignant epithelial cells and is downregulated via a β-catenin/Tcf-4 axis in cancer cells able to grow in an anchorage-independent manner [11]. Further evidence corroborating a tumor-suppressive role of DAPK2 comes from hematological malignancies, where its stable over-expression enhances all-trans retinoic acid (ATRA)-induced granulocytic differentiation of leukemic cell lines [12]. Consistently, in breast cancer, DAPK2 has emerged as a key mediator in paclitaxel-induced apoptosis, since its depletion by miR-520h results in drug resistance [13]. 

Non-coding RNAs contribute to fine-tune the expression of the main actors of the apoptotic response, and interfering with their function has provided encouraging results in preclinical models of cancer [14]. In this scenario, we performed a functional screening with a LNA-based anti-miRs library in human colorectal cancer cells, to identify pro-tumoral microRNAs. We discovered a novel oncogenic microRNA in colorectal cancer and showed that LNA-mediated targeting of miR-1285 triggers a powerful cytotoxic effect. Finally, we propose a role for DAPK2 as a mediator of cell cycle arrest and apoptosis in colorectal cancer cells treated with an anti-miR-1285 inhibitor.

## 2. Results

### 2.1. Genome-Wide Functional Screening with microRNA Inhibitors Identifies miR-1285-3p as a Potential Oncogene in Colorectal Cancer

**Figure 1:**
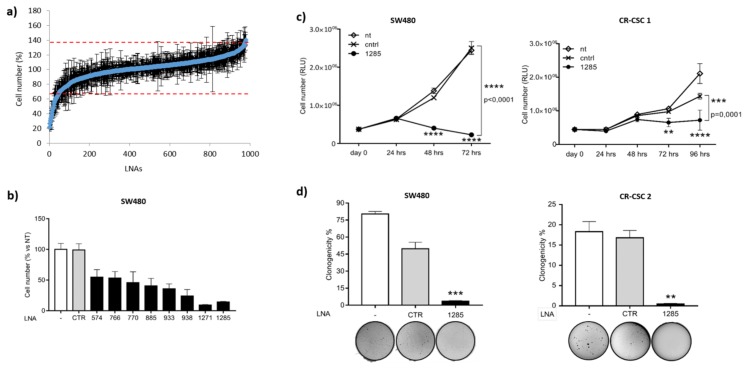
MiR-1285 inhibition impairs cell survival/proliferation and clonogenic potential in colorectal cancer cell lines and patient-derived cells. (**a**) Diagram showing results of the functional anti-miR screening in SW480 cells (906 LNAs tested). The viable cell number was evaluated 72 h after transfection by Cell Titer-Glo luminescent cell viability assay and normalized to the average of all samples, excluding controls. Each dot represents a single LNA. Values above and below two-fold the standard deviation (red dashed lines) were considered as significant. The experiment was performed in triplicate and the mean values ± SD were plotted. (**b**) Validation of the screening results. Cell number was evaluated by Cell Titer Glo assay 72 h after transfection of SW480 cells with the indicated LNAs at 10 nM and percentages versus the untreated sample (-) are reported (mean ± SEM). (**c**) Growth curves of both SW480 and colorectal cancer stem cells (CR-CSC 1) untreated (nt) and following treatment with LNA-1285 or control LNA at 5 nM and 25 nM, respectively. Statistical analysis was performed by ANOVA test for unpaired groups (** *p* < 0.01, *** *p* < 0.001, **** *p* < 0.0001). (**d**) Soft agar colony formation assays in both SW480 and colorectal cancer stem cells (CR-CSC 2) untreated (-) and upon transfection with LNA-1285 or control LNA at 25 nM. The graph shows the percentage of plated cells that gave rise to colonies (mean ± SD). *p*-values were calculated by Student’s *t* test (** *p* < 0.01, *** *p* < 0.001).

To identify the microRNAs responsible for oncogenic features in colorectal cancer, we exploited an unbiased high-content screening, following a previously optimized workflow [15,16]. Specifically, we challenged SW480 cells with a library containing 866 LNA inhibitors of mature human miRNAs annotated in miRBase version 12.0 (www.miRBase.org) and 40 other potential, currently not annotated, miRNAs. We monitored the effect of miR inhibition on cell viability and/or proliferation by Cell Titer Glo assay (Promega). Data were normalized to the average of all samples, excluding controls, and values exceeding two-fold the standard deviation were considered significant for further analyses. Interestingly, knockdown of 45 microRNAs significantly impaired cell viability/proliferation (Figure 1a). To validate the screening results in a small-scale setting, we selected eight LNAs among the top hits. Consistently, all the miR inhibitors tested confirmed their efficacy in reducing CRC cell viability in SW480 and HCT-116 cells (Figure 1b and Figure S1a). We focused on miR-1285-3p (hereafter miR-1285), whose inhibition resulted in a strong reduction in cell viability/proliferation in both cell lines. 

Next, we validated the pro-tumoral role of miR-1285 in different CRC cell lines and on patient-derived primary cells grown as tumorspheres and enriched in cancer stem cells (CR-CSCs). Patient-derived colorectal cancer stem cells (CR-CSCs) were first isolated on the basis of CD133 expression and proved to induce tumors in mice that resembled the original malignancy [17], thus representing a reliable pre-clinical model. Although whether colorectal tumors arise from this sub-population is still a matter of debate, it is widely accepted that CR-CSCs are responsible for cancer recurrence, dissemination and therapy failure, candidating them to be the best therapeutic target to pursue [18,19]. 

Of note, the assayed cells harbor different mutational profiles in the EGFR signaling pathway that recapitulate the spectrum of CRC tumors found in patients (Table S1). Neutralization of miR-1285 strongly impaired cell proliferation in all the cellular models tested, as shown by growth curves and anchorage-independent growth assays (Figure 1c,d and Figure S1b–e). 

### 2.2. Inhibition of miR-1285 Induces Cell Cycle Arrest and Apoptosis in Colorectal Cancer

**Figure 2:**
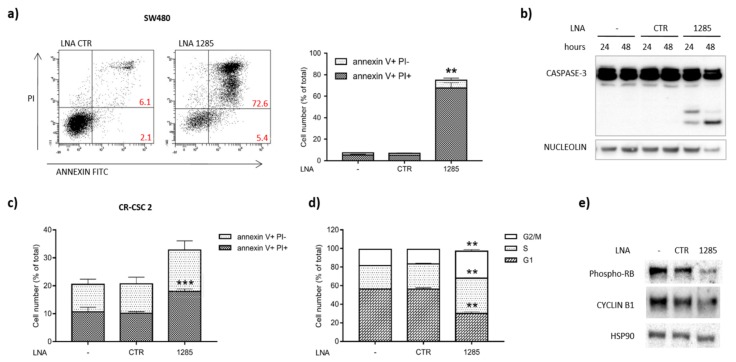
Targeting miR-1285 by an LNA-based anti-miR induces apoptosis and cell cycle arrest. (**a**) PI/Annexin V staining in SW480 cells: the dot plot shows a representative experiment, whereas the mean values of two different experiments are plotted in the graph (mean ± SEM). (**b**) Total and cleaved Caspase-3 levels were assayed by Western blot; nucleolin was detected as a loading control. (**c**) PI/Annexin V staining in colorectal cancer stem cells. Mean values of three independent experiments are shown (mean ± SEM). (**d**) FACS analysis of cell cycle in SW480 cells untreated (-) and transfected with control LNA or LNA-1285. The graph shows the percentage of cells in the different phases of the cell cycle. Error bars represent SEM. (**e**) Western blot showing reduced levels of phospho-RB and Cyclin B1 upon miR-1285 depletion in CR-CSC 2. HSP90 levels are shown as loading control. The (-) symbol indicates the untreated control. *p*-values were calculated by Student’s *t* test (** *p* < 0.01, *** *p* < 0.001).

In order to dissect the molecular events governed by miR-1285 we deepened our analyses by different functional assays. MiR-1285 inhibition was sufficient to elicit apoptosis, as shown by PI/Annexin V staining and confirmed by PARP-1/CASPASE-3 activation (Figure 2a–c). We observed programmed cell death induction upon miR-1285 deprivation in CRC cell lines and primary CSCs (Figure S2a–c), thus sustaining the oncogenic role of miR-1285 in CRC.

Interestingly, at early time points following treatment with low doses of LNA-1285, we observed alterations of the cell cycle, with a significant accumulation of cells in the G2/M phase (Figure 2d). We further confirmed the perturbation of the cell cycle in miR-1285-depleted cells by the reduction of RB phosphorylation and Cyclin B1 levels (Figure 2e). Altogether, these data suggest a specific role of miR-1285 in cell cycle control and cell death, with miR inhibition leading CRC cells to stall and, consequently, to massive apoptosis. 

**Figure 3:**
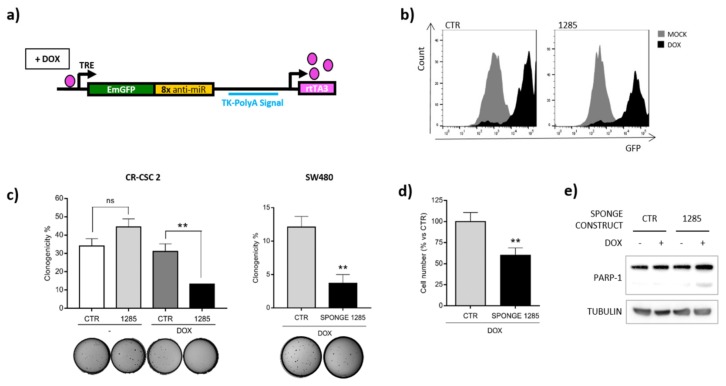
MiR-1285 blockade by a lentiviral sponge construct phenocopies the anti-survival effect of LNA-mediated inhibition. (**a**) Schematic representation of the doxycycline-inducible expression construct for the miR-1285 sponge (Tet-ON system): TRE = tetracycline response element, rtTA3 = reverse tetracycline transactivator 3. The transactivator binds to the TRE in the presence of doxycycline. (**b**) FACS analysis of GFP expression upon doxycycline treatment of CR-CSC 2 cells transduced with control virus or anti-miR-1285 sponge. (**c**) Soft agar colony formation assays in both cancer stem cells and SW480. Cells were transduced with the indicated lentiviral constructs and treated with doxycycline. Mean values of one or two independent experiments performed in triplicate are shown for CR-CSCs and SW480 cells, respectively. The graph shows the percentage of plated cells that gave rise to colonies (mean ± SEM). (**d**) Assessment of cell number by Cell Titer Glo assay in transduced primary cancer stem cells (CR-CSC 2) following doxycycline administration. Mean values of two independent experiments performed in quadruplicate are shown (mean ± SEM). (**e**) Detection of PARP-1 cleavage by Western blot in transduced SW480 cells treated with doxycycline. The (+) and (-) symbols indicate the presence or absence of doxycycline. *p*-values were calculated by Student’s t test (*ns* = not significant, ** *p* < 0.01).

To rule out non-specific effects of LNA-1285-3p in CRC cells, we developed a sponge construct to knock down miR-1285. Namely, we cloned a cassette containing eight repetitions of the miR-1285 binding site downstream of the GFP Open Reading Frame (ORF) in the inducible lentiviral vector pTRIPZ (Open Biosystems) (Figure 3a). In the presence of doxycycline, the transactivator rtTA3 binds to the TRE promoter, thus inducing GFP expression (Figure 3b). The miR-1285 binding sites, located in the GFP 3′UTR, function as a sponge and titer down miR levels in transduced cells. While LNA inhibitors determine a strong and virtually permanent inhibition of miR levels, miR modulation obtained by sponge constructs is generally milder and it was not always sufficient to irreversibly trigger the apoptotic cascade. Albeit to a lesser extent compared to LNA-1285 treatment, infection of both SW480 and CR-CSCs with this construct significantly impaired cell proliferation and capacity to generate colonies in soft agar, leading to apoptosis as indicated by PARP-1 cleavage (Figure 3c–e).

**Figure 4:**
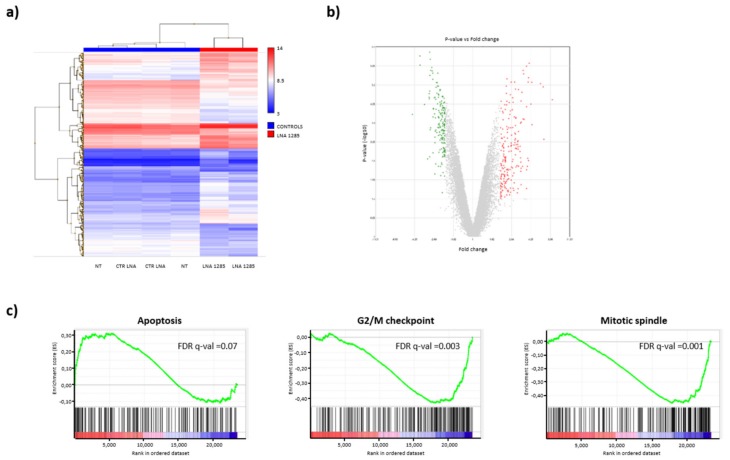
Transcriptome analyses upon LNA-1285 treatment. (**a**) Heat map showing unsupervised hierarchical clustering of all modulated genes in LNA-1285-treated SW480 cells compared to control cells (untreated + control LNA). (**b**) Volcano plot representing all modulated genes (upregulated in red and downregulated in green). (**c**) Gene set enrichment analysis (GSEA) for differential gene expression in miR-1285-depleted cells shows significant modulation of genes related to apoptosis and cell cycle arrest. FDR q-val < 0.05 are reported for each gene set.

We next exploited transcriptome analyses to identify the most affected pathways upon miR-1285 depletion and better dissect the miR-1285 interactome (Whole Transcript Expression Arrays, HuGene 1.0 St, Affymetrix, Santa Clara, CA, USA). Total RNA was extracted from SW480 cells, transfected with either control LNA or LNA-1285 and processed for microarrays. We found 225 upregulated and 172 downmodulated genes upon miR-1285 depletion (Figure 4a,b). Corroborating our observations, gene set enrichment analyses (GSEA, [20,21]) pointed out a significant induction in the expression of apoptotic genes upon miR-1285 knockdown, while genes involved in G2/M checkpoint and in mitotic spindle assembly were suppressed, supporting cell cycle arrest (Figure 4c).

### 2.3. DAPK2 Is a Novel Direct Target of miR-1285 in Colorectal Cancer

In order to identify key mediators of the above-mentioned phenotypes among miR-1285 putative targets, we performed in silico analyses, taking advantage of the algorithms TargetScan, DIANA tools and microRNAs.org. In particular, we focused on positive regulators of apoptosis and integrated microRNA target predictions by TargetScan (release 7.2) with the list of pro-apoptotic genes. By crossing the result of this analysis with the list of genes that were upregulated ≥1.5-fold by gene arrays upon miR-1285 depletion, we identified two genes: TNFRSF9 and BCL2L11. The former is a member of the death receptor superfamily, which mediates extrinsic apoptosis. However, TNFRSF9 is mainly expressed in immune cells and is an inducible T cell costimulatory receptor, primarily expressed on activated CD4+ and CD8+ T cells. Based on this background, we discarded TNFRSF9 from our analyses. The latter, BCL2L11, is a BH3-only protein directly inducing apoptosis, but it displays the lowest score of predicted efficacy of targeting of all the selected hits (Table S2). Provided that microRNAs modulate their targets post-transcriptionally, often no modulation at the RNA level occurs and bona-fide targets may not be identified by transcriptome profiling. For this reason, we focused on the high-score hits and selected Death-Associated Protein Kinase 2 (DAPK2) for further analyses (Table S2).

**Figure 5:**
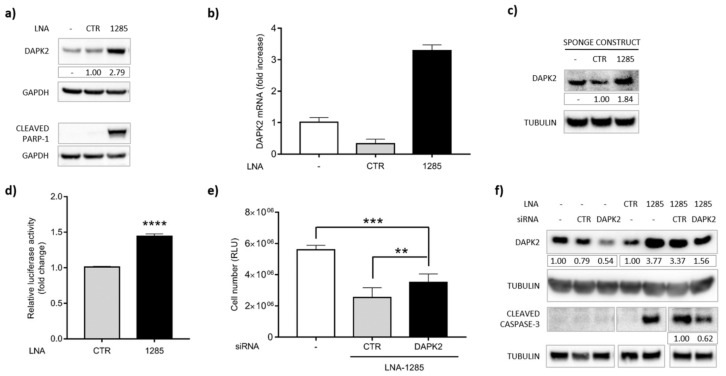
DAPK2 is a direct target of miR-1285 and mediates apoptosis induction in miR-1285 depleted cells. (**a**) Western blot showing an increase in DAPK2 levels and concomitant PARP-1 cleavage in SW480 cells upon transfection with LNA-1285 at 25 nM for 24 h and in untreated cells (-). The same protein samples were loaded in two different gels. GAPDH levels are shown as a loading control. The densitometric analysis is reported as fold change compared to the sample treated with control LNA. (**b**) RT-qPCR for DAPK2 in SW480 cells untreated (-) or transfected with the indicated LNA at 25 nM for 72 h. (**c**) Western blot showing the increase in DAPK2 levels in primary CR-CSC 2 transduced with the anti-miR-1285 lentiviral sponge construct, control construct or untreated (-), upon doxycycline treatment. (**d**) Luciferase activity in HeLa cells transfected with DAPK2 3′UTR in combination with control or anti-miR-1285 LNA. The graph shows the normalized luciferase activity of three independent experiments performed in quadruplicate (mean ± SEM). (**e**,**f**) Cell Titer-Glo assay and Western blot show that down-modulation of DAPK2 by RNA interference can rescue the LNA-1285 apoptotic effect in SW480 cells. The (-) symbol indicates untreated cells. (**e**) Cell number was measured 72 h upon transfection with the indicated molecules (mean ± SEM). (**f**) Western blotting showing DAPK2 modulation in SW480 cells after the indicated treatments. Double transfection of anti-DAPK2 siRNA together with LNA-1285 almost restores the DAPK2 levels of the control cells, counteracting LNA-mediated induction. CASP-3 cleavage is reduced to ~60%. TUBULIN was used as the protein-loading control. Numbers indicate DAPK2 levels as the fold change relative to the control-treated cells, and cleaved CASP-3 levels as the fold decrease relative to the LNA-1285/control siRNA-treated cells. The values were normalized to the TUBULIN signal, as measured by densitometric analyses. The second gel image was cropped in order to display the samples in an order different from the one of the loading. Tubulin levels are shown as a loading control. *p*-values were calculated by Student’s *t* test (** *p* < 0.01, *** *p* < 0.001).

DAPK2 3′UTR contains several putative miR-1285 binding sites, three of which were identified by at least two of the abovementioned algorithms (Figure S3). DAPK2 belongs to the DAP-kinase family of proteins, which positively mediate apoptosis and autophagy. Despite DAPK2 mRNA not being significantly modulated in the transcriptome analyses, we observed increased expression of DAPK2, at both the mRNA and protein level, upon treatment with LNA-1285 (Figure 5a,b and Figure S4a). Further, DAPK2 accumulation was associated with PARP-1 cleavage already after 24 h of treatment (Figure 5a). 

We confirmed the induction of the DAPK2 protein also by sponge-mediated miR-inhibition. Namely, transduction of the sponge-1285 in primary cells triggered DAPK2 protein enrichment compared to the control cells (Figure 5c), thus supporting the inverse correlation between miR-1285 and DAPK2. Further, we exploited the luciferase reporter assay to demonstrate the direct binding of miR-1285 to DAPK2 3′UTR. To this end, the 3′UTR of DAPK2 was cloned downstream of the *Renilla* luciferase gene (rLuc) in the psiCHECK-2 vector (Promega). Upon transfection, we measured rLuc expression in the presence of LNA-1285 or control LNA. rLuc values were normalized to firefly luciferase levels as transfection control. As shown in Figure 5d, miR-1285 knockdown was sufficient to sustain rLuc induction, indicating that DAPK2 is negatively controlled by and is a bona fide direct target of miR-1285.

### 2.4. DAPK2 Mediates miR-1285 Oncogenic Role 

To define the role of this new miR-1285 target, we performed knockdown of DAPK2 by siRNAs (Figure S4b). We observed that DAPK2 silencing partially restored cell viability of SW480 cells depleted for miR-1285 (Figure 5e). Notably, the concomitant transfection of anti-DAPK2 siRNA and LNA-1285 did not completely knockdown the DAPK2 protein, but almost restored the same DAPK2 levels of untreated cells, counteracting the LNA-mediated induction. Such modulation of DAPK2 relieved the pro-apoptotic effect of LNA-1285, as shown by reduced levels of cleaved CASPASE-3 (Figure 5f). Altogether, these data confirm that DAPK2 mediates the anti-tumoral effect of miR-1285 targeting in CRC. The partial rescue of cell viability and cleaved CASPASE-3 obtained by siRNA is consistent with the cooperative action of DAPK2 with other, yet unidentified, miR-1285 targets. 

**Figure 6:**
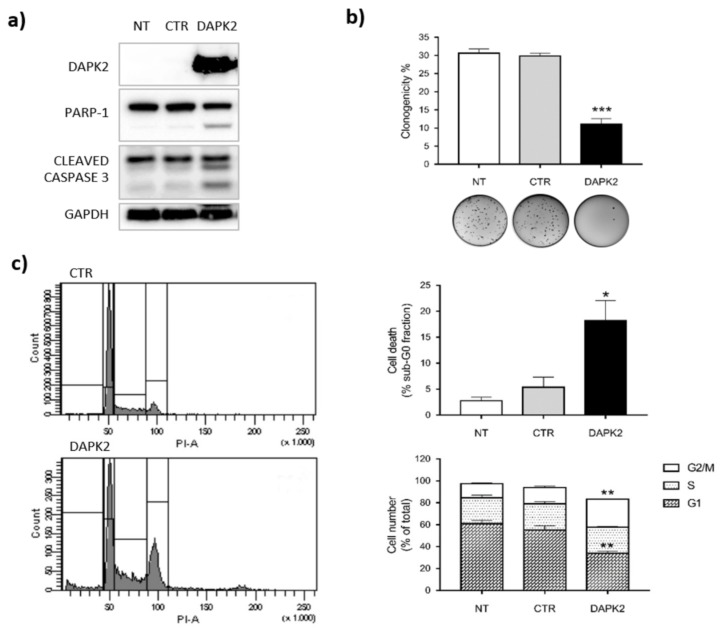
DAPK2 over-expression recapitulates both the apoptosis induction and the cell cycle arrest observed upon miR-1285 inhibition. SW480 cells were transduced with a lentiviral construct for ectopic expression of DAPK2 and compared to their counterparts either harboring the empty vector (CTR) or untreated (NT). (**a**) Expression levels of DAPK2, PARP-1 and cleaved Caspase-3 in the indicated samples. GAPDH levels were assessed as a loading control. (**b**) Soft agar colony formation assay. The graph shows the percentage of plated cells that gave rise to colonies. (**c**) Cell cycle analysis showing cell populations in sub-G0, G1, S and G2/M phases: left panel shows one representative experiment, whereas the histograms show the mean values of four independent experiments. Error bars represent SEM values and *p*-values were calculated by Student’s t test (* *p* < 0.05, ** *p* < 0.01, *** *p* < 0.001).

To further verify the role of DAPK2 among other miR-1285 targets, we ectopically over-expressed it by lentiviral transduction and measured the capacity of CRC cells to grow in an anchorage independent manner, cell cycle and apoptosis. Consistently with our previous findings, DAPK2 over-expression was sufficient to recapitulate the main phenotypes observed upon miR-1285 depletion in SW480 cells and in CR-CSCs (Figure 6 and Figure S4c). 

Indeed, the clonogenic capacity of DAPK2 over-expressing cells was significantly impaired compared to control cells (Figure 6b). Further, FACS analyses revealed that DAPK2 over-expression leads to a block in G2/M phase and the concomitant increase of cells in sub-G0 phase, indicative of apoptosis (Figure 6c and Figure S4c). We confirmed apoptotic induction upon DAPK2 over-expression by PARP-1 and CASPASE-3 cleavage (Figure 6a). Altogether, these data support the role of DAPK2 in cell cycle control and apoptosis induction and show how inhibition of DAPK2 expression represents a major mechanism underlying miR-1285 oncogenic function in CRC. 

## 3. Discussion

Although the advent of anti-EGF receptor (EGFR)-targeted therapies has significantly improved patients’ prognosis, effective treatment of advanced colorectal cancer is still an unmet need. In fact, only a subset of patients can benefit from EGFR-blocking drugs, since mutations leading to constitutive KRAS activation, occurring in 40% of cases, confer resistance [22]. This scenario urges the search for novel key targets to expand the therapeutic options. The deepening in the molecular bases of cancer biology and the advances of medical biotechnologies led to theorize the concept of “smart drugs”, aimed at selectively killing cancer cells by targeting key mediators of the tumorigenic process. MicroRNAs are endogenous non-coding small RNAs that fine-tune protein expression. The connection between aberrant accumulation of oncogenic microRNAs and tumor growth and spread suggests a new strategy to fight cancer by interfering with miR function. The great potential of miR targeting relies on the ability of microRNAs to simultaneously regulate target genes belonging to different pathways. Therefore, by hitting a single microRNA we may concurrently overcome multiple escape strategies developed by transformed cells and hijack the miR-mediated machinery to push cancer cells toward non-tumoral settings. This approach turned out to be effective in several tumor models. To name a few, in vivo delivery of oligonucleotides directed against miR-424, miR-135b, miR-21 and miR-182 showed significant anti-tumor activity in mouse models of prostate and colorectal cancer, multiple myeloma and melanoma liver metastasis, respectively [23,24,25,26]. Among all chemically modified anti-miR oligonucleotides, LNAs show a strong binding affinity and resistance to nuclease degradation. Notably, anti-miR-122 LNA was successfully employed in the treatment of hepatitis C virus (HCV) infection in non-human primates [27] and passed a phase II clinical trial in 36 patients with chronic HCV-1 infection (ClinicalTrials.gov identifier: NCT01200420) [28]. On this exciting background, LNA-based inhibitors represent the best candidate molecules for successful approaches to be translated in vivo and, hopefully, in clinics. 

In this study, we performed an unbiased screening of LNA-based miR-inhibitors and identified miR-1285-3p as a novel oncogene in CRC. By loss of function experiments, we demonstrated how miR-1285 sustains cell proliferation and controls the escape from apoptosis in CRC cells, as confirmed by transcriptome analyses on miR-1285-depleted cells. We found that miR-1285 neutralization triggers a strong anti-tumoral response also in patient-derived cancer stem cells (CR-CSCs), thus suggesting the potential effectiveness of this approach in reducing tumor burden. In fact, CSCs represent the resistant sub-population that fuels tumor growth and dissemination to distant organs [18,19]. 

To date, only a few reports have described the role of miR-1285-3p in cancer, showing that it can act as both tumor-suppressor and onco-miR, depending on the context and tumor type [29,30,31]. This apparent controversial, dual role of microRNAs has been already reported as a consequence of differential gene expression patterns, determining a tumor-specific interactome for a given microRNA.

Here, we identified DAPK2 as the main mediator of miR-1285 in CRC. Indeed, DAPK2 ectopic expression is sufficient to reproduce the onco-suppressive program observed upon miR-1285 targeting, while DAPK2 knockdown can partially rescue miR-depleted cells from apoptosis. Further, for the first time we provide evidence on cell cycle regulation by DAPK2. Although DAPK2 onco-suppressive role has been characterized, little is still known about its mechanisms of action [9,10]. The N-terminal region of DAPK2, spanning the kinase domain and the CaM regulatory domain, shows a high degree of sequence similarity with DAPK1, the founding member of this protein family, whereas the C-terminal tail is required for self-dimerization. Only a few interactors of DAPK2 have been identified so far [32,33,34]. Accordingly, just a limited number of DAPK2 substrates is currently known: myosin II regulatory light chain, the mTOR binding partner raptor [35], the autophagy receptor protein p62/SQSTM [36] and the core autophagic machinery protein Beclin-1 [37]. DAPK2 function is tightly controlled at multiple layers, suggesting a relevant function for this protein that needs to be under strict and fine regulation. Autophosphorylation on Ser308 within the CaM regulatory domain prevents both binding to Ca^2+^/Calmodulin and homodimerization, thus inhibiting DAPK2 catalytic activity [38]; a similar inhibitory effect is achieved through binding of 14-3-3 to the C-terminal tail [32]. On the contrary, during metabolic stress, AMPK-mediated phosphorylation on Ser289 activates DAPK2 by functionally mimicking Calmodulin binding [37]. Moreover, DAPK2 is regulated at the transcriptional level by recruitment of several transcription factors to its promoter, such as Sp1, E2F1, KLF6 [39] and the myeloid master regulators PU.1 and C/EBPα [40]. Post-transcriptional regulation of DAPK2 expression by non-coding RNAs (ncRNAs) has been reported in the recent literature [41,42,43]. Here, we demonstrate that DAPK2 expression in colorectal cancer is controlled by miR-1285, thus providing further insight into the post-transcriptional layer of DAPK2 regulation. 

## 4. Materials and Methods

### 4.1. Cell Culture

All human colorectal cancer cell lines (SW480, HCT-116 and HT-29) were derived from the American Type Culture Collection (ATCC, Manassas, VA, USA). SW480 were grown in RPMI 1640 with L-glutamine (Lonza, Basel, Switzerland) supplemented with 10% heat-inactivated fetal bovine serum (PAA, Pasching, Austria). HCT-116 and HT-29 were cultured in McCoy’s 5A (Modified) Medium GlutaMAX Supplement (Gibco, Thermo Fisher Scientific, Waltham, MA, USA) supplemented with 10% heat-inactivated fetal bovine serum. Colorectal cancer stem cells were isolated from tumor specimens and grown in suspension as tumorspheres in a serum-free medium supplemented with 20 ng/mL EGF and 10 ng/mL FGF-2 (Peprotech, London, UK), as previously described [17]. At every passage and before any transfection procedure, cancer stem cells were dissociated in smaller tumorspheres or single cells, respectively, with TrypLE Express Enzyme (1X) phenol red (Gibco, Thermo Fisher Scientific, Waltham, MA, USA) according to the manufacturer’s instructions. Human embryonic kidney 293T cells (ATCC, Manassas, VA, USA) were cultured in Iscove’s Modified Dulbecco’s Medium (IMDM, Euroclone, Pero, Italy) supplemented with 10% heat-inactivated fetal bovine serum and 1% L-glutamine.

### 4.2. DNA Constructs and Transduction/Transfection Procedures

A cassette containing EmGFP only or EmGFP-anti-miR-1285 (4x) maxi-sponge (containing 8 miR-binding sites) was subcloned from a pcDNA6.2 vector (Invitrogen, Thermo Fisher Scientific, Waltham, MA, USA) into a pTween vector. The cassette in the pTween vector was PCR-amplified with primers containing AgeI and MluI restriction sites for subcloning into the pTRIPZ lentiviral inducible empty vector (Open Biosystems, Huntsville, AL, USA). Next, the PCR product was cloned downstream of the CMV promoter fused to the tetracycline response element (TRE) in the pTRIPZ vector upon digestion with AgeI and MluI (New England Biolabs, Ipswich, MA, USA). The lentiviral CAD-G Whiz (CGW) vector expressing DAPK2 was kindly provided by prof. M.P. Tschan (University of Bern, Bern, Switzerland). Both pTRIPZ and CGW vectors were used to produce third generation lentiviral particles. Briefly, 293T cells were transfected with any of the aforementioned transfer vectors, the envelope vector pMD2G and the packaging vector pCMV (pPAX) using the Calcium Phosphate transfection method. Forty-eight hours post-transfection, cell supernatants were collected, 0.45 µm-filtered and 100-fold concentrated using the Lenti-X Concentrator (Clontech, Mountain View, CA, USA). Viral titer was calculated by measuring by flow cytometry the number of GFP-positive cells upon transduction with serial dilutions of the virus, assuming that for a yield of GFP-positive cells <20% each cell was transduced by no more than one lentiviral particle. SW480 and cancer stem cells were transduced with MOI = 20 and MOI = 30, respectively. PTRIPZ-transduced cells underwent selection with 2 µg/mL (SW480) and 3 µg/mL (cancer stem cells) puromycin, whereas CGW-transduced cells were freshly used for the experiments as they underwent cell cycle arrest and apoptosis shortly after transduction. 

Full-length DAPK2 3’UTR was PCR-amplified from genomic DNA extracted from human blood cells of a healthy donor, using the following primers for cloning into psiCHECK2 vector (Promega, Madison, WI, USA): DAPK2 3’UTR-XhoI-for 5′-GATCTCGAGACTGGCCTGACCTG CAGTGG-3′ and DAPK2 3’UTR-NotI-rev 5′-ATAGCGGCCGCGGGTTGGTTTCCTCTGAGGC-3′. Transfection experiments were performed using DharmaFECT 2 Transfection Reagent (Dharmacon, Lafayette, CO, USA) for all ATCC colorectal cancer cell lines, whereas HiPerFect Transfection Reagent (Qiagen, Thermo Fisher Scientific, Waltham, MA, USA) was used for cancer stem cells, following manufacturer’s instructions. A combination of 4 different siRNAs (Smart-Pool, Dharmacon, Lafayette, CO, USA) were used at 50 nM final concentration. LNAs (Exiqon, Woburn, MA, USA) were used at 5, 10, 25 or 50 nM final concentration. 

### 4.3. LNA Library Screening

The screening of the LNA library (Exiqon, Woburn, MA, USA) was performed as previously described [16]. Briefly, SW480 cells (2 × 10^3^ cells/well) were seeded in 96-well plates. DharmaFECT 2 Transfection Reagent (Dharmacon, Lafayette, CO, USA) was diluted in Optimem medium (Thermo Fisher Scientific, Waltham, MA, USA), seeded on LNAs and incubated for 15–20 min at room temperature. The transfection mixture was then added to the cells at the final LNA concentration of 25 nM. These conditions were formerly set up to ensure ~90% transfection efficiency. The percentage of transfected cells was monitored with a control siRNA against NF1b mRNA (40 nM), and verified by Real-Time PCR. As a positive control, the siRNA against PLK-1 was added on empty wells of the library plates. Cancer cells were assayed 72 h post-transfection with the CellTiter-Glo Luminescent Cell Viability Assay (Promega, Madison, WI, USA) on a Beckman DTX 880 plate luminometer (Beckman Coulter, Brea, CA, USA). The relative viability was calculated and normalized to the average of all samples, excluding positive controls. The experiment was performed in triplicate. Values below and above twofold the standard deviation were considered significant and were used in further analyses.

### 4.4. Anchorage-Independent Assay (Soft Agar Colony Formation Assay)

SW480 and cancer stem cells were seeded in 24-well plates and transfected with 25 nM LNA-1285 or non-targeting LNA-CTR. Twenty-four hours post-transfection, cells were trypsinized (or Tryple-dissociated) and counted. A total of 500 cells was resuspended in 1.5 mL semisolid culture medium supplemented with 0.3% Agar Noble (Difco, Kansas City, MO, USA) and plated on 1,5 mL solidified culture medium supplemented with 0.6% Agar Noble. Colonies formation was assessed after two weeks (for ATCC cell lines) or four weeks (for cancer stem cells) upon crystal violet (Fluka, St. Gallen, Switzerland) staining and manual counting with a microscope. Stable sponge-1285-overexpressing cells (both SW480 and cancer stem cells) were seeded in a 24-well plate in culture medium supplemented with 0.5 µg/mL doxycycline. Upon 48 h doxycycline treatment, cells were harvested and counted, and a fraction of the cell suspension was FACS-analyzed for GFP expression, in order to test for the efficacy of doxycycline treatment in inducing the expression of the sponge cassette. Of the leftover cells, 500 cells were used for the colony formation assay and colonies were counted after four weeks.

### 4.5. Cell Proliferation and Apoptosis Detection

For proliferation assays, 2 × 10^3^ cells/well were seeded in 96-well plate and transfected with 25 nM LNA-1285 or non-targeting LNA-CTR. Stable sponge-1285-overexpressing cancer stem cells were pre-treated with 0.5 µg/mL doxycycline. At the indicated time points, three wells for each condition were analyzed. Growth curves were generated by evaluating cell number with CellTiter-Glo Luminescent Cell Viability Assay (Promega, Madison, WI, USA), following manufacturer’s instructions. 

Apoptosis was measured with the Apoptosis Detection Kit (MBL International, Woburn, MA, USA). Briefly, cells were seeded in 24-well plates and transfected with 50 nM LNA; cells were harvested 48 h (for ATCC cell lines) or 96 h (for cancer stem cells) post-transfection, counted and stained with Annexin V-FITC and PI (5 µg/mL) and analyzed using a BD FACSCanto Cytometer (Becton Dickinson, San Jose, CA, USA). For stable DAPK2-overexpressing cells, the percentage of apoptotic cells was assessed five days upon lentiviral transduction by detecting the sub-G0 fraction following incubation with Nicoletti buffer (0.1% sodium citrate pH 7.4, 0.1% NP40, 9.65 mM NaCl, 200 mg/mL RNAse A, 50 mg/mL PI) and FACS analysis.

### 4.6. Cell Cycle Analysis

Stable DAPK2-overexpressing SW480 and cancer stem cells were analyzed 4–6 days post-transduction, respectively. Alternatively, SW480 cells were treated with 5 or 10 nM LNA for 24 h. Briefly, cells were incubated with Nicoletti buffer and the percentage of cells in G1, S and G2/M phase was assessed by FACS analysis.

### 4.7. Immunoblotting

Whole cell protein extracts (lysis buffer: 30 mM Tris-HCl pH 7.5, 150 mM NaCl, 2 mM KCl, 2 mM EDTA, 5% Glycerol, 0.1% Triton X-100 and 1× Protease Inhibitor Cocktail (Sigma-Aldrich, Merck, Darmstadt, Germany) were quantified by BCA assay (Pierce, Thermo Fisher Scientific, Waltham, MA, USA), separated onto NuPAGE 4%–12% polyacrylamide gels (Invitrogen, Thermo Fisher Scientific, Waltham, MA, USA) and blotted on nitrocellulose membranes (Amersham, Little Chalfont, United Kingdom). Membranes were hybridized with polyclonal antibodies anti-PARP-1 (#9542 Cell Signaling Technology, Danvers, MA, USA), anti-cleaved PARP-1 (#9541 Cell Signaling Technology, Danvers, MA, USA), anti-Caspase-3 (#9665 Cell Signaling Technology), anti-cleaved Caspase-3 (#9661 Cell Signaling Technology, Danvers, MA, USA), anti-DAPK2 (#07-1229 Millipore, Burlington, MA, USA), anti-phospho-Rb [ser780] (#3590 Cell Signaling Technology, Danvers, MA, USA) anti-Cyclin B1 (#4135 Cell Signaling Technology, Danvers, MA, USA). Monoclonal antibodies anti-Actin (#CP01, Calbiochem, Merck, Darmstadt, Germany), anti-α-Tubulin (#T5168 Sigma- Aldrich, Merck, Darmstadt, Germany) and anti-Nucleolin (#sc-8031 Santa Cruz Biotechnology, Santa Cruz, CA, USA) or polyclonal antibody anti-GAPDH (#G9545 Sigma-Aldrich, Merck, Darmstadt, Germany) were used as loading controls. Bands were visualized and quantified with FluorChem E System (ProteinSimple, San Jose, CA, USA). 

### 4.8. Luciferase Reporter Assay

In luciferase experiments, HeLa cells were seeded in 96-well plates (4 × 10^3^ cells/well) and transfected with 40 ng of Firefly luciferase vectors (empty psiCHECK-2 or psiCHECK-2-DAPK2 3’UTR wt) and 4 ng of *Renilla* luciferase vector (pRL-TK, Promega) together with 50 nM LNAs. Lipofectamine 2000 (Invitrogen) was used as a transfection reagent (0.2 µL/well). The activity of both Firefly and *Renilla* luciferases was measured 48–72 h post-transfection using the Dual Luciferase Assay kit (Promega) and the luminescence plate reader Victor-X3 (PerkinElmer, Waltham, MA, USA). Transfection efficiency was normalized by calculating the ratio firefly/*Renilla*. The experiment was performed 3 times in quadruplicate.

### 4.9. RT-qPCR Analysis

For mRNA analysis, SW480 cells were treated with 25 nM LNA-1285 for 72 h and total RNA was purified with TRIZOL Reagent (Invitrogen, Thermo Fisher Scientific, Waltham, MA, USA) and reverse-transcribed with random primers (N6, Roche, Basel, Switzerland) and M-MLV RT (Invitrogen) after DNase-I treatment (RQ1 DNase, Promega, Madison, WI, USA). Real-time PCR was performed with SensiMix SYBR Hi-ROX (Bioline, London, United Kingdom) using a gene-specific Taqman probe assay for human DAPK2 (Applied Biosystems, Thermo Fisher Scientific, Waltham, MA, USA). Samples were run on a StepOne Real-Time PCR System (Applied Biosystems, Thermo Fisher Scientific, Waltham, MA, USA) according to standard procedures. Human GAPDH was used as endogenous control. 

## Supplementary Materials

The following are available online at https://www.mdpi.com/1422-0067/21/7/2423/s1.
