# Supplementary figures and images for: miR-1285-3p Controls Colorectal Cancer Proliferation and Escape from Apoptosis through DAPK2
Source: Int J Mol Sci. 2020 Mar 31;21(7):2423. doi: 10.3390/ijms21072423 (PMC7177834; doi:10.3390/ijms21072423)

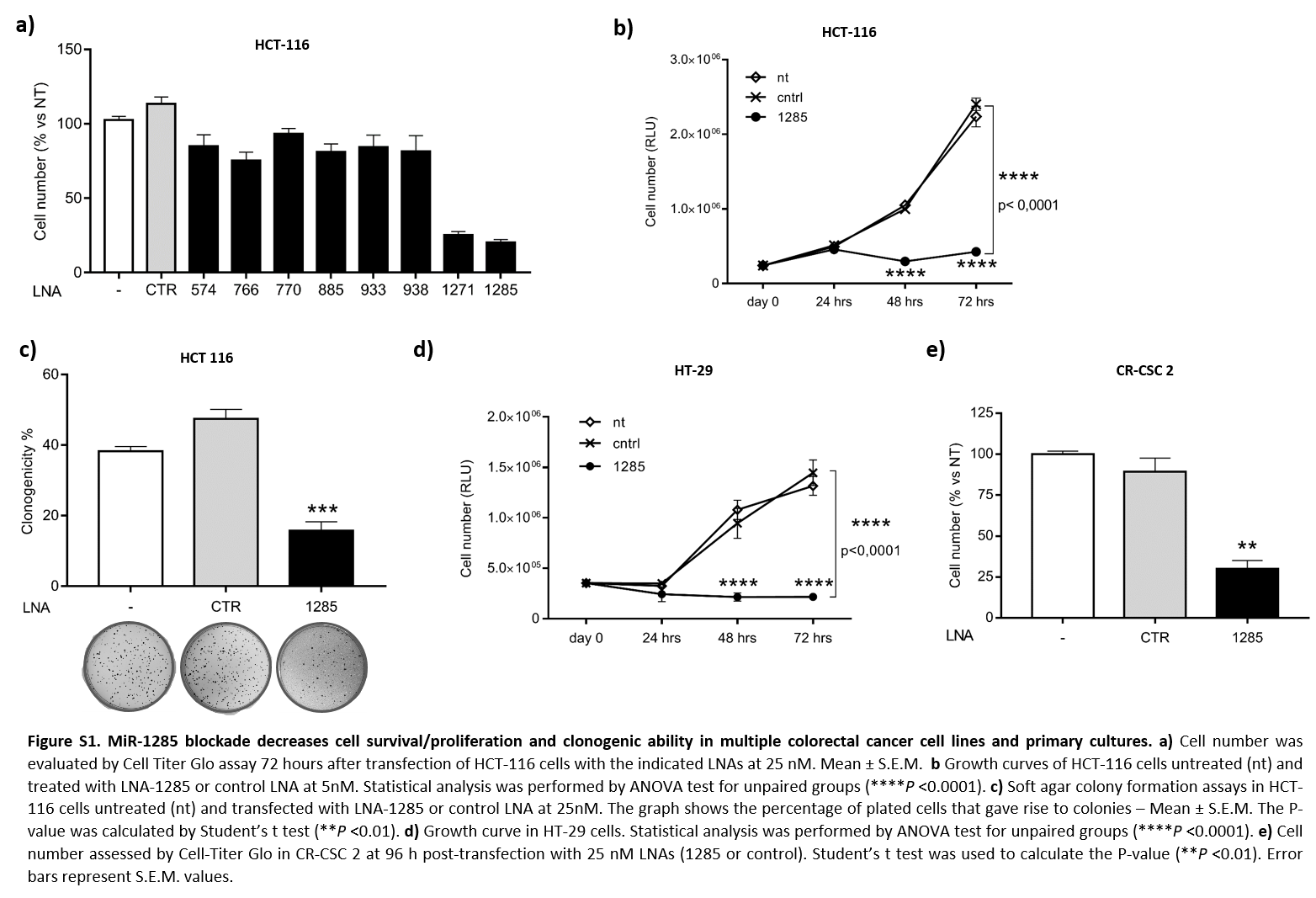

Supplement: Supplementary file 1 [file ijms-21-02423-s001.zip › ijms-719258supplementary-F/Figure S1 con legenda.tif]

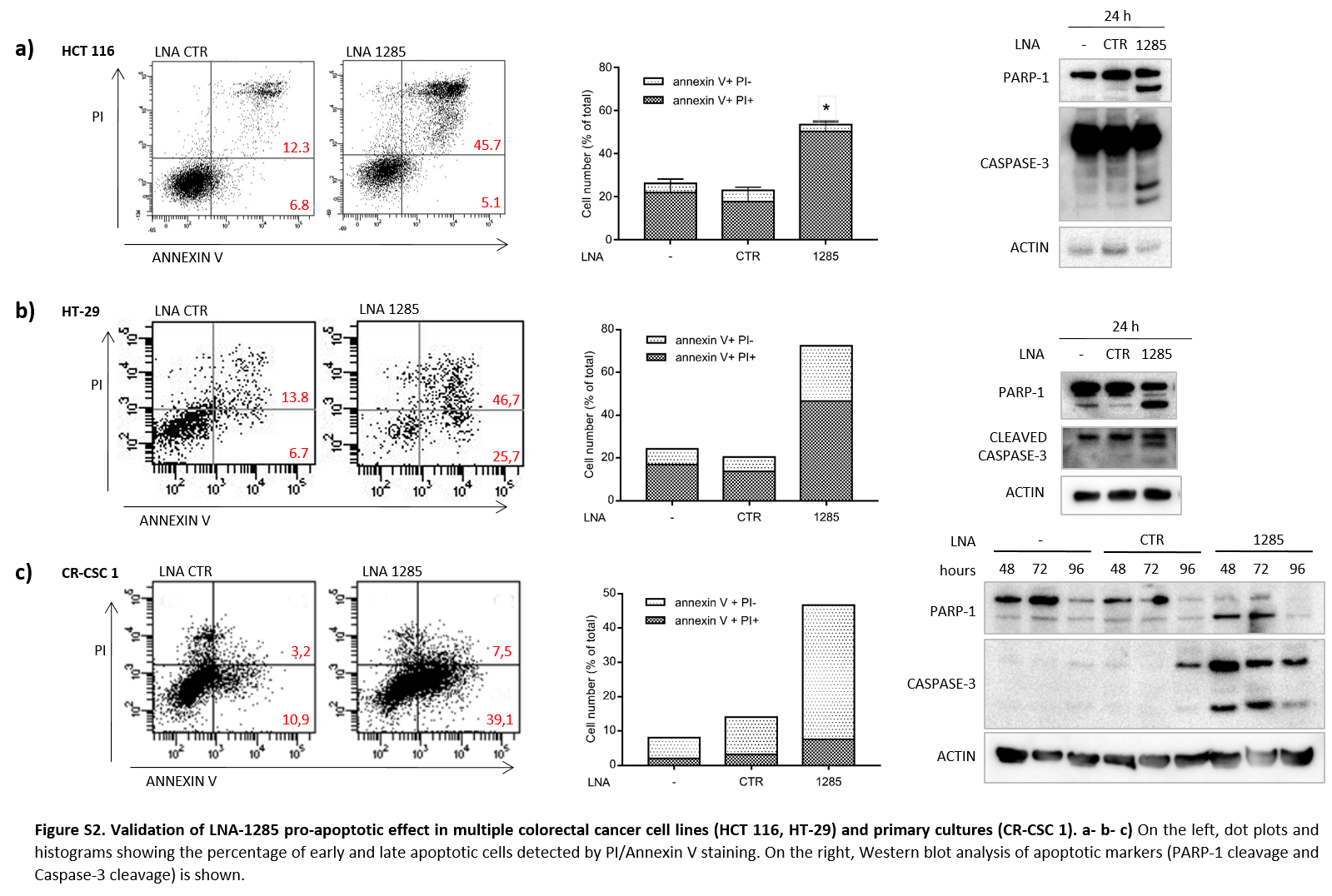

Supplement: Supplementary file 1 [file ijms-21-02423-s001.zip › ijms-719258supplementary-F/Figure S2 con legenda.tif]

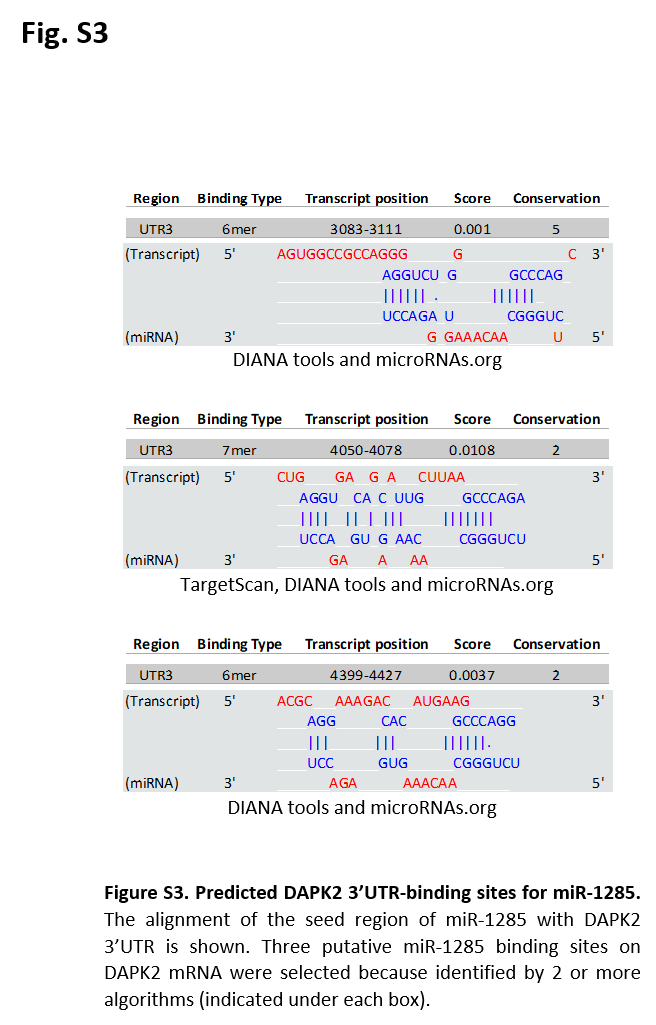

Supplement: Supplementary file 1 [file ijms-21-02423-s001.zip › ijms-719258supplementary-F/Figure S3 con legenda.tif]

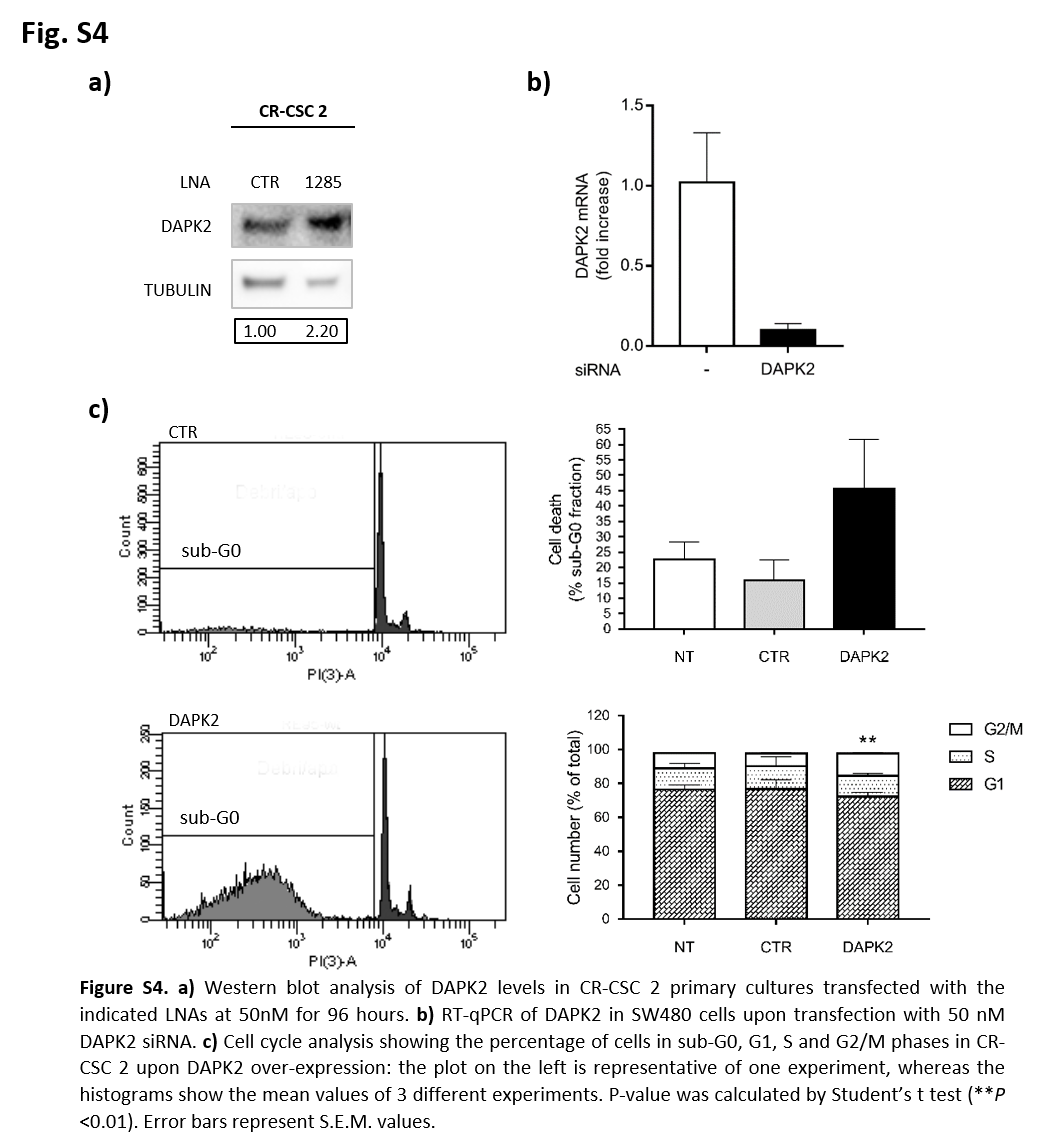

Supplement: Supplementary file 1 [file ijms-21-02423-s001.zip › ijms-719258supplementary-F/Figure S4 con legenda.tif]

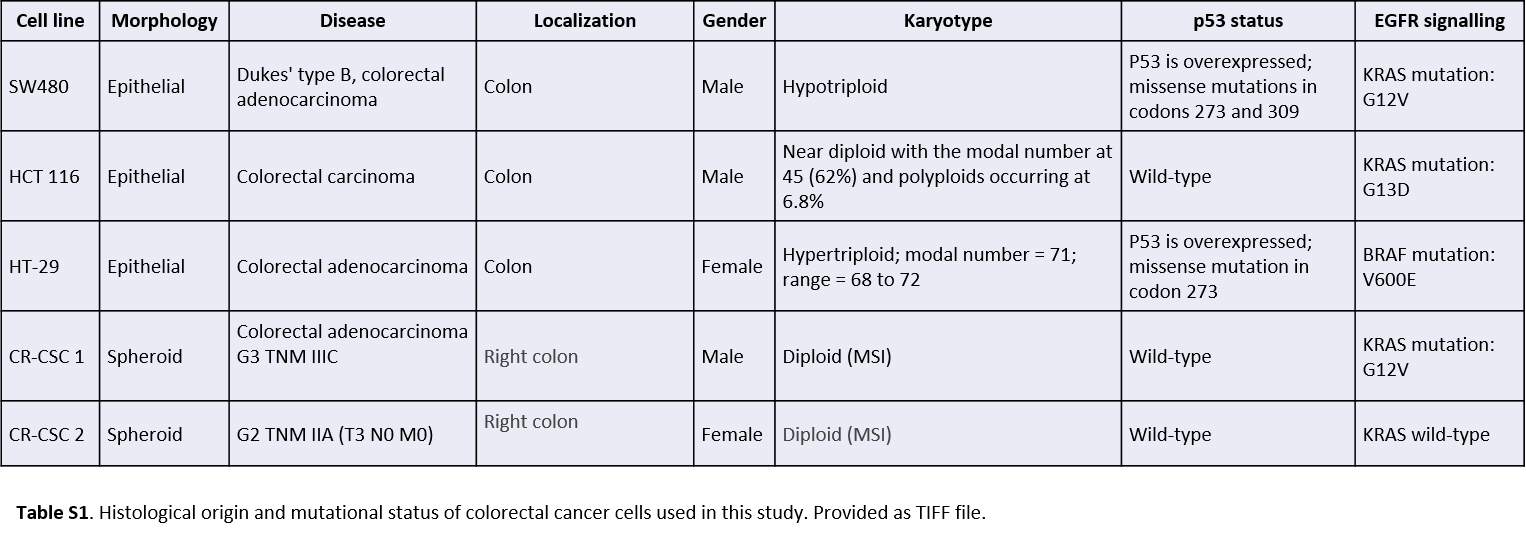

Supplement: Supplementary file 1 [file ijms-21-02423-s001.zip › ijms-719258supplementary-F/Table S1.tif]

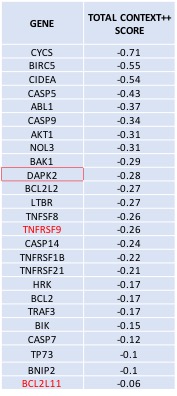

Supplement: Supplementary file 1 [file ijms-21-02423-s001.zip › ijms-719258supplementary-F/Table S2.jpg]
